# Function and Molecular Mechanism of Circhomer1 in Myogenesis

**DOI:** 10.3390/ijms26136264

**Published:** 2025-06-28

**Authors:** Zonggang Yu, Kaiming Wang, Bohe Chen, Jingwen Liu, Wenwu Chen, Haiming Ma

**Affiliations:** 1 College of Animal Science and Technology, Hunan Agricultural University, Changsha 410128, China; zgyu333@stu.hunau.edu.cn (Z.Y.); 15116529648@stu.hunau.edu.cn (K.W.); 13847652015@stu.hunau.edu.cn (B.C.); ljw@stu.hunau.edu.cn (J.L.); cww1242646778@stu.hunau.edu.cn (W.C.); 2 Key Laboratory of Livestock and Poultry Resources (Pig) Evaluation and Utilization, Ministry of Agriculture and Rural Affairs, Changsha 410128, China; 3 Yuelushan Laboratory, Changsha 410128, China

**Keywords:** myogenesis, circHOMER1, ceRNA, encoding

## Abstract

Skeletal muscle is one of the largest tissues in the body. It is of great significance to analyze the molecular mechanism of skeletal muscle development for the further study of meat quality improvement and muscle diseases. CircRNA has been reported to be involved in many biological processes, but further research is needed in skeletal muscle. In this study, we detected the authenticity, stability, and spatio-temporal expression characteristics of circHOMER1 and its effect on the proliferation, apoptosis, and differentiation of muscle cells, and analyzed its possible molecular mechanism. The results showed that circHOMER1 exists in the skeletal muscle of the Ningxiang pig, is more stable than linear RNA, and is significantly upregulated in adipose tissue and during the early growth of myoblasts. In terms of function, overexpression of circHOMER1 significantly promoted the expression levels of proliferation marker genes and proteins and significantly increased the EdU positive cell rate, optical density (OD) value (at 450 nm), and proportion of S-phase cells. Overexpression of circHOMER1 also significantly promoted the expression levels of apoptosis marker genes and proteins and significantly increased the proportions of cells in Q2 (with late apoptosis) and Q3 (with early apoptosis). Overexpression of circHOMER1 significantly inhibited the expression levels of differentiation marker genes and proteins, significantly inhibited the differentiation index, and decreased the proportion of 5-nucleus muscle fibers. Conversely, opposite results were obtained after circHOMER1 interference. In terms of molecules mechanism, subcellular localization analysis showed that circHOMER1 was mainly distributed in cytoplasm, and mechanism analysis showed that circHOMER1 participated in myoblast development by forming a 4-element interaction network with 4 miRNAs, 2 lncRNAs, and 20 mRNAs, and possibly regulated myoblast development by encoding 79 amino acids. To sum up, we verified that circHOMER1 promoted the proliferation and apoptosis of myoblasts and inhibited their differentiation. It may regulate the development of myoblasts through ceRNA or by encoding small peptides. These results provided a reference for the regulation mechanism of muscle development and the breeding of Ningxiang pigs.

## 1. Introduction

With the improvement in their living standards, people are no longer satisfied with having enough to eat but pay more attention to the quality and safety of food. Ningxiang pig is an excellent indigenous pig breed in China, which has delicious meat, tolerance for rough feeding, and strong stress resistance [1], but its growth rate is slow. Skeletal muscle development is the key limiting step affecting meat yield, and the growth speed significantly affects the age of slaughter. After the muscle is fully developed, fat is deposited in the muscle to form marbling; therefore, the development of skeletal muscle is also an important factor affecting meat quality. The development of skeletal muscle is regulated by key genes, non-coding RNA, and other transcriptional regulators. MYOD [2], MYHC, MYOG [3,4], MYF5 [5], and MEF2 [6,7,8] have been reported to affect the process of muscle proliferation and differentiation. Similarly, miRNAs [9,10], such as miR-1, miR-133, and miR-206, and lncRNAs [11,12,13], such as lncRAM, LncRNA-Six1, and Lnc-231, have also been reported to be involved in regulating muscle development. CircRNA is also involved in the growth, regeneration, and differentiation of skeletal muscle [14,15,16]. However, there are few reports on the regulation of skeletal muscle development by circRNA in Ningxiang pigs.

circHOMER1 was identified in 2013 [17], but it did not attract much attention initially and was not reported in human diseases until 2020. Hsa_circ_0006916 (circHOMER1) has been shown to influence amyloid β-induced neuronal damage by targeting miR-217/HOMER1 [18]. CircHOMER1 inhibited porcine adipogenesis through the miR-23b/SIRT1 axis [19]. It was identified as a marker of prenatal drinking in mice [20]. CircHOMER1 can aggravate oxidative stress, inflammation, and extracellular matrix deposition in human interstitial cells induced by hyperglycemia through the miR-137/SOX6 signal axis and can be used as a molecular target for diabetes treatment [19]. The bidirectional competitive interaction between circHOMER1 and Homer1b in the frontotemporal cortex regulates brain function [21]. It has been found that circHOMER1 is specifically expressed in the cerebral cortex of females with amyloidosis, which further affects the progression of Alzheimer’s disease [22]. After binding with miR-1332, circHOMER1 promoted the growth and invasion of hepatocellular carcinoma cells by enhancing CXCL6 expression [23]. CircHOMER1 was highly expressed in Alzheimer’s disease and frontotemporal degeneration, which suggested that it may affect the progression of the disease [24]. Knockout of circHOMER1 inhibited the expression of Bbc3 and improved the neuronal damage induced by METH [25]. The circHOMER1/miR-138-5p/HEY1 axis played an important role in inhibiting the proliferation and aerobic glycolysis of colorectal cancer cells [26]. Further analysis showed that the length of circHOMER1 in human diseases is 522 nt and that it contains four exons. These findings show that research on circHOMER1 is mainly focused on human diseases, but research on its role in livestock and poultry production is still in its infancy, highlighting the urgent need for further study.

CircRNA relieves the inhibition of miRNA on target genes by competitively binding with the target gene of miRNA. In this study, *HOMER1* was obtained by overlapping the key genes of WGCNA and ceRNA with the host genes of differentially expressed circRNA, and its corresponding cricRNA was Chr02_64288819_64298085. Therefore, we further verified its function in myoblasts and explored its potential molecular mechanism. The aim of this study is to provide a reference for the mechanism research of muscle development in Ningxiang pigs.

## 2. Results

### 2.1. Characteristics Analysis of circHOMER1

**Figure 1:**
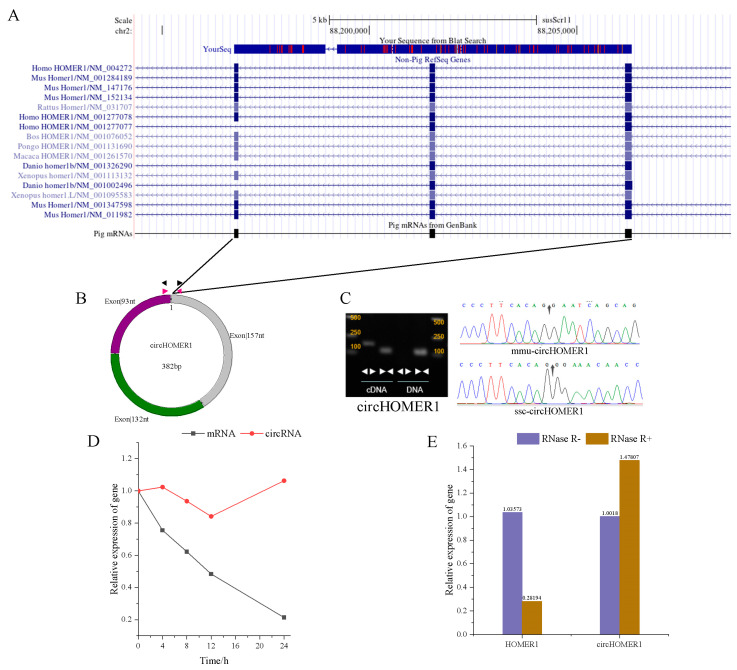
Characteristic analysis and cyclization verification of circHOMER1 (Chr02_64288819_64298085). The exon of *HOMER1* gene in human and mouse was compared through UCSC website (**A**), model plot (**B**) and cyclization verification of exonic circHOMER1 by gel plot and sanger sequencing (**C**). (**D**,**E**) were the relative expression of mRNA and circRNA of Actinomycin D-treated cells and RNase R-treated RNA, respectively.

After sequencing, Chr02_64288819_64298085 was identified as an exon circRNA, which was located on chromosome 2 of the Ningxiang pig. Comparing the circRNA sequence with the reference genome of susScr11, it was found that it came from the *HOMER1* gene, including three exons of 157 nt, 132 nt and 93 nt, with a total length of 382 nt, and was highly homologous to human and mouse genes. Therefore, this study termed it circHOMER1 (Figure 1A,B). Furthermore, PCR, nucleic acid agarose gel electrophoresis, and Sanger sequencing were used to verify whether it was cyclized. It was found that divergent primers did not amplify effectively in gDNA, but divergent primers amplified bands in cDNA and convergent primers amplified bands in both DNA types, which indicated that circHOMER1 was formed by back splicing (Figure 1C). The reverse splicing site was identified by Sanger sequencing of the products amplified using divergent primers, which indicated that it really existed. The stability of circRNA was tested using actinomycin D and RNase R. It was found that with the extension of actinomycin D treatment time, the expression of circRNA remained basically unchanged, but the expression of mRNA decreased significantly. After RNase R-treated RNA, the mRNA expression of circHOMER1 did not decrease, but the mRNA expression of linear *HOMER1* decreased significantly (Figure 1D,E). These results show that circHOMER1 is more stable than *HOMER1*.

### 2.2. Homologous Transformation of circHOMER1

CircHOMER1 was found to be homologous circRNA in humans, mice and pigs via homologous transformation analysis (Table 1). Evolutionarily conserved sequences may have similar functions. Therefore, circHOMER1 may have the same function in homologous species.

### 2.3. Detection of Expression Pattern of circHOMER1

The spatio-temporal expression analysis of circHOMER1 for Ningxiang pigs showed that its expression level was obviously different. During weaning, the expression of circHOMER1 increased significantly in fat, liver, lung and kidney and decreased significantly in the longissimus dorsi muscle (Figure S1A). Compared with heart tissue, the nursing period was significantly higher in kidney and adipose tissue, and there was no significant difference in other tissues (Figure S1B). At the early stage of fattening, the expression level in liver and fat increased significantly, and it increased significantly in the spleen and kidney, but there was no significant difference between the lung and muscle (Figure S1C). The expression of circHOMER1 in the liver was significantly higher than that in heart tissue and increased significantly in the spleen, lung, kidney and fat, but there was no significant difference between muscle and heart (Figure S1D). Compared with the weaning period, the expression of circHOMER1 was significantly higher in the nursing period and later fattening period (Figure S1E).

The expression level of circHOMER1 in myoblasts in different stages of in vitro culture was detected. The expression of circHOMER1 in the early growth stage (16 h) increased significantly (Figure S1F). In the differentiation stage, the expression level increased significantly at the pre-differentiation stage (culture in differentiation medium for 2d and 4d), reaching *p* < 0.0001, and post-differentiation stage (culture in differentiation medium for 6d and 8d) decreased, but it was still significantly higher than that at the 0-day differentiation stage (Figure S1G).

### 2.4. Detection Overexpression and Interference Efficiency for circHOMER1

After transfection of the circHOMER1 plasmid and siRNA, the efficiency was detected via RT-qCPR. The results showed that the best transfection scheme of overexpression plasmid was 1:2 (transfect 2.5 μg of overexpression plasmid with 5 μL of Lipofectamine 2000), and the overexpression efficiency was more than 54-times (Figure S2A). By designing two siRNAs, setting two concentrations of 50 nM and 100 nM, and using 5 μL of Lipofectamine 2000 for transfection, it was found that the best scheme for knock-down was to use 5 μL of Lipofectamine 2000 for transfection of 100 nM siRNAs, and the knock-down rate was below 75% (Figure S2B).

### 2.5. circHOMER1 Promotes the Proliferation of Myoblasts

**Figure 2:**
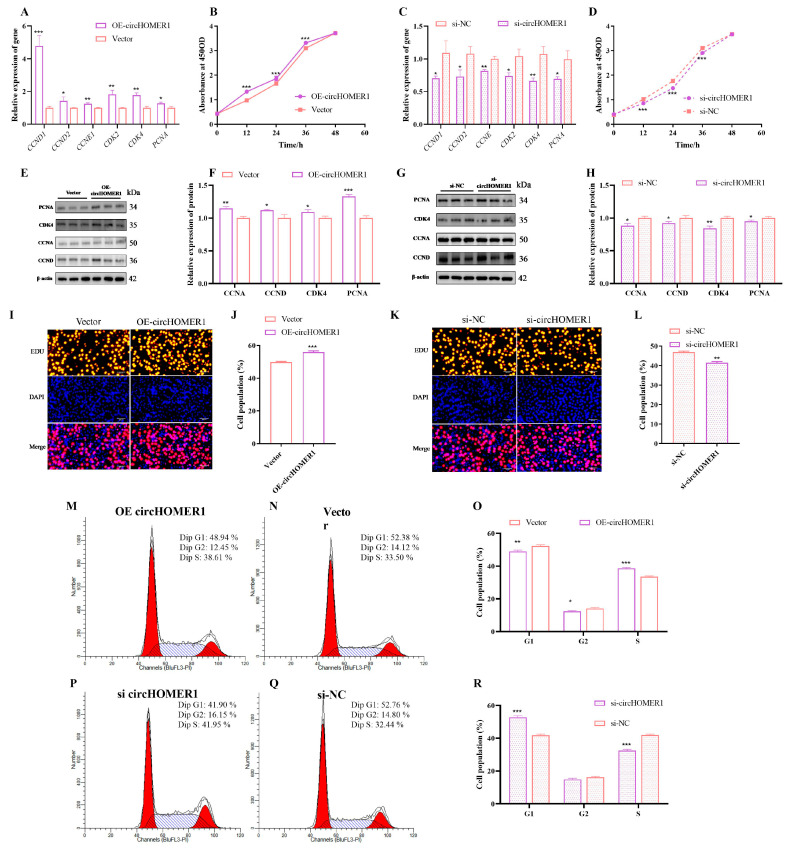
circHOMER1 promotes the proliferation of myoblasts. After overexpression or siRNA circHOMER1, RT-qCPR, WB, CCK8, EdU and flow cytometry were used to detect the expression of proliferation marker genes and proteins (**A**,**C**,**E**–**H**), cell absorbance at 450nm (**B**,**D**), the proportion of positive cells stained with EdU (**I**–**L**) and the proportion of cells in different cell cycles (**M**–**R**) to evaluate the effects on myoblast proliferation. The scale of EdU picture was 200 μm. *n* = 3, statistical testing was depicted as two-tails, unpaired *t*-test; * *p* < 0.05, ** *p* < 0.01, *** *p* < 0.001.

Compared with the control group, overexpression of circHOMER1 significantly increased the expression level of the proliferation marker gene (Figure 2A) and significantly increased the absorbance at 450 nm (Figure 2B) and the EdU-positive cell rate (Figure 2I,J). The expression level of proliferation marker protein in the overexpression group was significantly increased (Figure 2E,F). The proportion of cells in the G1 phase (prophase of synthesis) in the overexpression group was significantly lower than that in the control group, and the proportion of cells in the S phase (prophase of synthesis) was significantly higher than that in the control group. These results indicated that overexpression of circHOMER1 significantly promoted cell proliferation (Figure 2M–O).

The expression of the proliferation marker gene (Figure 2C), absorbance at 450nm (Figure 2D) and the proportion of edU-stained cells (Figure 2K,L) decreased significantly after the myoblast knocked down circHOMER1. The expression level of proliferating protein (Figure 2G,H) and the proportion of S-phase cells (Figure 2P–R) in the knock-down group decreased significantly, and the proportion of G1-phase cells in the knock-down group increased significantly (Figure 2P–R).

### 2.6. circHOMER1 Promotes Myoblast Apoptosis

**Figure 3:**
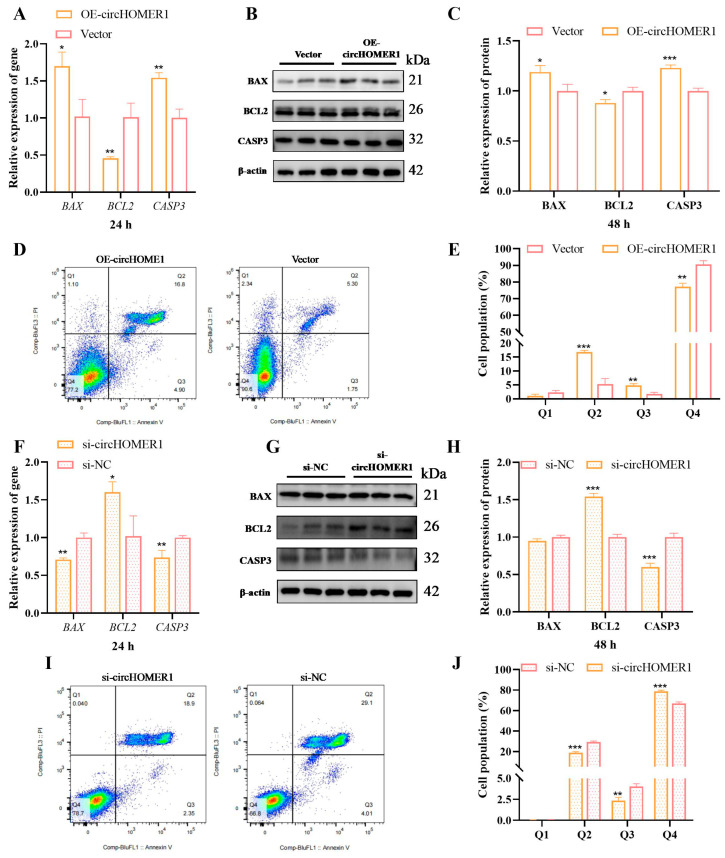
circHOMER1 promotes myoblast apoptosis. After overexpressing or interfering with circHOMER1 in myoblasts, the effects on myoblast apoptosis were detected by RT-qCPR (**A**,**F**), WB (**B**,**C**,**G**,**H**) and flow cytometry (**D**,**E**,**I**,**J**). n = 3, statistical testing was depicted as two-tails, unpaired *t*-test; * *p* < 0.05, ** *p* < 0.01, *** *p* < 0.001.

The effect of circHOMER1 on the apoptosis process of cells was detected based on the overexpression and knock-down of circHOMER1. The results showed that the expression level of apoptosis marker genes and proteins in the overexpression group increased significantly, while the expression of anti-apoptosis marker genes and proteins decreased significantly (Figure 3A–C). Flow apoptosis analysis showed that the proportion of cells in the Q2 region (the proportion of late apoptotic cells) and Q3 region (the proportion of early apoptotic cells) in the overexpression group was significantly higher than that in the control group, and the proportion of cells in the Q4 region was significantly lower (Figure 3D,E). The results obtained after knocking down circHOMER1 were contrary to those obtained after overexpressing circHOMER1.

### 2.7. circHOMER1 Inhibits Myoblast Differentiation

**Figure 4:**
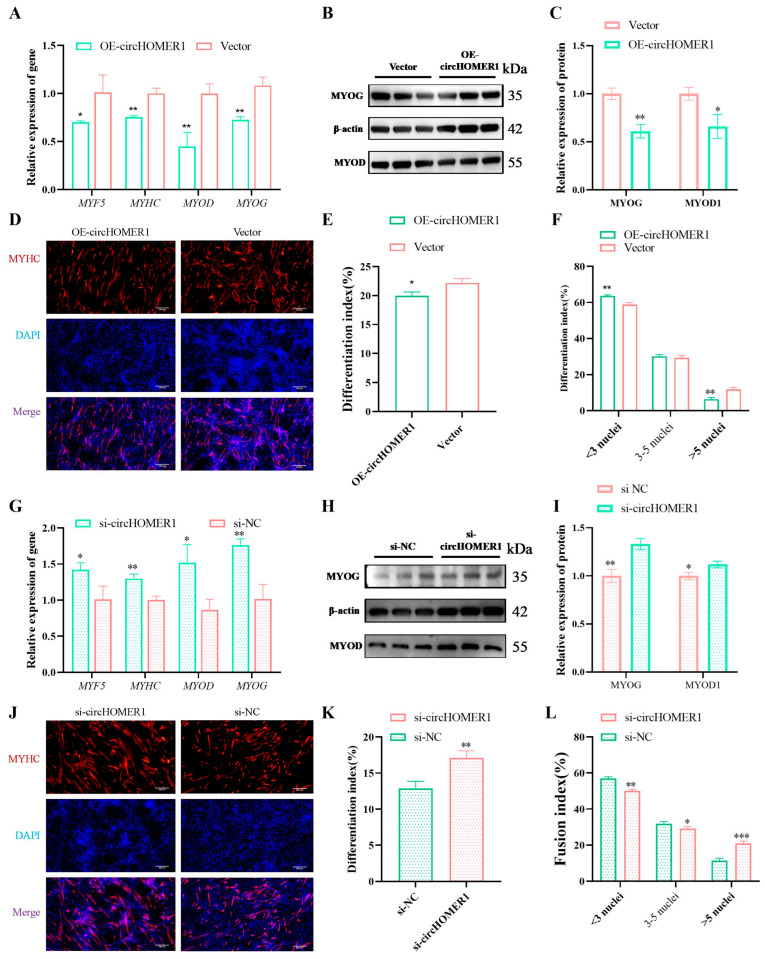
circHOMER1 inhibits myogenic differentiation. After transfection with circHOMER1 overexpression plasmid or siRNA, the expression of differentiation marker gene (**A**,**G**) and protein (**B**,**C**,**H**,**I**) and the percentage of differentiated cells (**D**,**E**,**J**,**K**) and fused cells (**F**,**L**) were detected by RT-qCPR, WB and IF. The scale of IF picture was 200 μm. *n* = 3, statistical testing was depicted as two-tails, unpaired *t*-test; * *p* < 0.05, ** *p* < 0.01, *** *p* < 0.001.

To detect the effect of circHOMER1 on myoblast differentiation, after the overexpression plasmid of circHOMER1 was transfected into the myoblast, the expression level of the differentiation marker gene was detected after 5 days of induction differentiation. The results showed that the mRNA level and protein level of *MYHC*, *MYOD1*, *MYF5* and *MYOG* were significantly or extremely significantly decreased compared with the control group (Figure 4A–C). The differentiation index and fusion index were further tested, and it was found that overexpression of circHOMER1 significantly promoted the differentiation index and the percentage of myotubes below three nuclei and significantly decreased the percentage of myotubes above five nuclei (Figure 4D–F). The result of knocking down circHOMER1 was opposite to that of overexpressing circHOMER1.

### 2.8. Subcellular Localization Analysis of circHOMER1

**Figure 5:**
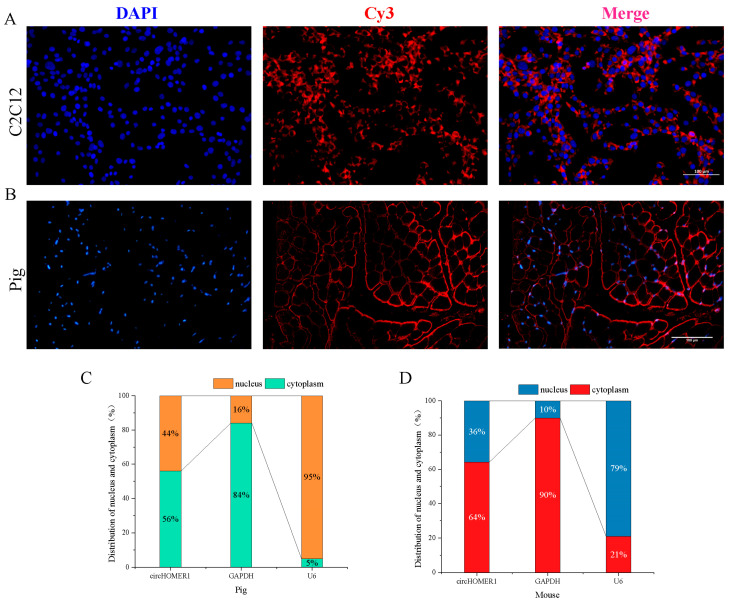
The subcellular localization of circHOMER1 in the myoblast (**A**) and the tissue section (**B**) of Ningxiang pig’s *longitu dorsi* muscle was detected by FISH, in which DAPI labeled the nucleus (blue) and Cy3 labeled circHOMER1 (red), and the scale bar was 100 μm. The nucleoplasm distribution of circHOMER 1 in mouse myoblasts (**C**) and LD (**D**) was detected by RT-qCPR. The gene internal references of the nucleus and cytoplasm were *U6* and *GAPDH*, respectively.

The function of circRNA is determined by its subcellular localization. In this study, the fluorescent probes of circHOMER1 in C2C12 cells (Figure 5A) and Ningxiang pig skeletal muscle (Figure 5B) were mainly distributed in cytoplasm. RT-qCPR detection of RNA isolated from C2C12 and Ningxiang pig skeletal muscle also showed that circHOMER1 was mainly distributed in the cytoplasm (Figure 5C,D).

### 2.9. Construction and Verification of ceRNA Network Mediated by circHOMER1

**Figure 6:**
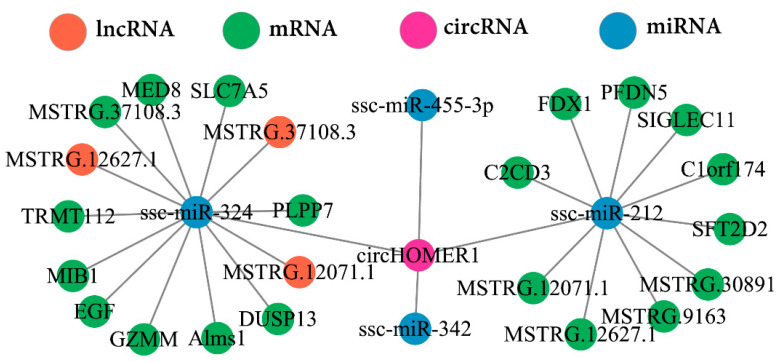
ceRNA network mediated by circHOMER1.

CircRNA located in the cytoplasm often competes with genes to bind to miRNA. Therefore, based on the previous research results, we constructed the ceRNA regulatory network of circHOMER1, which combines 4 miRNAs (ssc-miR-324, ssc-miR-455-3p, ssc-miR-212, ssc-miR-342), 3 lncRNAs and 20 mRNAs (Figure 6).

**Figure 7:**
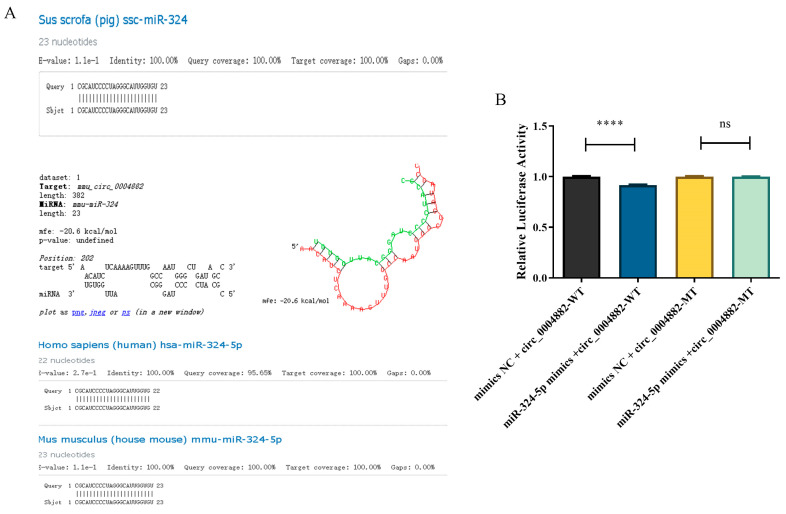
Conservative analysis of miR-324-5p and verification of ceRNA interaction. Conservation analysis of mir-324-5p in human, pig and mouse (**A**), and double luciferase report detection of mir-324-5p targeted binding with circHOMER1 (**B**). **** *p* < 0.0001, ns, not significant.

miR-324-5 was highly conserved in humans, mice and pigs (Figure 7A). Furthermore, the combination of circHOMER1 and miR-324-5p was verified by double luciferase report experiment (Figure 7B).

### 2.10. Prediction of Coding Ability of circHOMER1

**Figure 8:**
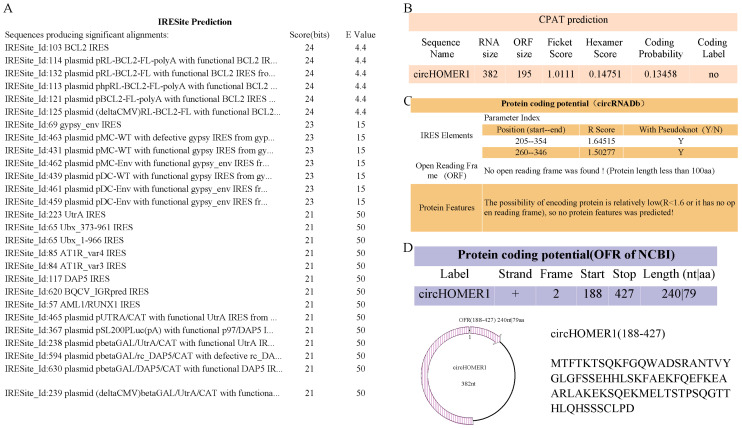
Protein coding potential analysis of circHOMER1. The analysis of protein coding potential of circHOMER1 by online software IRES (**A**), CPAT (**B**), circRNADb (**C**) and OFR (**D**), respectively.

circHOMR1 was not only distributed in the cytoplasm but also located in the nucleus. circRNA in the nucleus may also encode peptide fragments. Therefore, we also predicted the protein coding of circHOMER1 and found that there were many IRES in it, and CPAT analysis showed that there was no ORF in it (Figure 8A,B). circRNADb found IRES at 205–354 nt and cicRNA 250–346 nt, and the length of the amino acid (AA) encoded by OFR was no more than 100 (Figure 8C). The analysis of the protein coding potential showed that OFR with a length of 240 nt (located at 188–427 nt) could encode 79 amino acids (Figure 8D).

## 3. Discussion

Differential expression gene (DEG) analysis and WGCNA are commonly used to identify the key genes of a certain phenotype. *HOMER1*, a key gene related to skeletal muscle, was identified by overlapping analysis of WGCNA and DEG. *HOMER1* formed circHOMER1 through back splicing during post-transcriptional processing. CircHOMER1 was composed of three exons with a length of 382 nt (nucleotide). The authenticity of circHOMER1 in mice and pigs was verified by divergent primer PCR amplification and Sanger sequencing, and it was proved that circHOMER1 was more stable than linear *HOMER1* by RNase R and actinomycin D tests. Spatial–temporal expression profile analysis showed that the expression level of circHOMER1 in different stages of spatial expression analysis was significantly increased in fat, and the expression level of circHOMER1 in the LD of Ningxiang pig was significantly higher than that in the weaning period, the nursing period, and late fattening period. This suggests that circHOMER1 may play a vital role in the nursing period and the late fattening period. During the proliferation and differentiation of C2C12, the expression level of circHOMER1 increased significantly in the pro-phase of proliferation and in the differentiation stage, which suggested that it played an important role in regulating the proliferation and differentiation of myoblasts.

We detected circHOMER1’s effects on the proliferation, differentiation and apoptosis of myoblasts in C2C12 by gene overexpression/knock-down. In proliferation progress, overexpression of circHOMER1 promoted the proliferation of myoblasts and interfered with circHOMER1, inhibiting the proliferation of myoblasts, which indicated that circHOMER1 promoted the proliferation by regulating the expression of proliferation marker genes. In the process of apoptosis, overexpression of circHOMER1 promoted myoblast apoptosis, and knock-down of circHOMER1 inhibited myoblast apoptosis. In the process of differentiation, the differentiation index of myoblasts decreased significantly after overexpression of circHOMER1, and the proportion of 5-nuclear muscle fibers decreased significantly. After knocking down circHOMER1, the opposite result was obtained. To sum up, circHOMER1 can promote the proliferation and apoptosis of myoblasts and inhibit the differentiation process.

It has been reported that circRNA has different biological functions in different subcellular localizations [27]. circRNA located in the cytoplasm mainly regulates biological processes via ceRNA [17,28,29], while circRNA located in nucleus mediates biological processes by encoding small peptides [30,31]. Through subcellular localization detection, it was found that circHOMER1 was mainly distributed in the cytoplasm, which laid a foundation for it to regulate myogenic differentiation by ceRNA. Through joint analysis with the ceRNA network, it was found that circHOMER1 could bind to 4 miRNAs, and 4 miRNAs could bind to 3 lncRNAs and 20 mRNAs. Furthermore, the combination of circHOMER1 and miR-324-5p was verified via a double luciferase reporter assay. Knockdown of *Alms1* can increase proliferation markers in cardiomyocytes, the percentage of cardiomyocytes in the G2/M phase and the number of cardiomyocytes in cultured cells by 10% [32]. After overexpression of *HEGF*, the number of muscle stem cells (satellite cells) in muscle fibers increased significantly, which promoted myoblast regeneration [33]. Mind bomb-1 (Mib1) regulates α-actin 3 (ACTN3) through proteasome degradation dependence, which plays an important role in skeletal muscle maintenance [34]. Mib2 maintains the integrity of fully differentiated muscles through Notch and integrin-independent pathways, and this prevents their apoptotic degeneration [35]. The activity, differentiation and fusion of myoblasts were damaged by pharmacological inhibition and gene knockdown of *Slc7a5* [36]. All the above genes are the target genes of miR-324-5p, which also shows that different genes play different functions in the process of muscle development, and the function of some genes in muscle development has not been studied, so it needs further verification.

CircRNA located in the nucleus can encode small peptides. We predicted the encoding potential of circHOMER1 using various software and found that circHOMER1 can encode small peptides. CircHOMER1 regulates skeletal muscle development by encoding small peptides, which needs further verification.

## 4. Materials and Methods

### 4.1. Cell Culture and Samples

C2C12 was purchased from Anwei Biotechnology Co., Ltd. (Anweisci, Shanghai China). The cells were cultured in DMEM (Gibco, Carlsbad, CA, USA) containing 10% fetal bovine serum (Gibco, Carlsbad, CA, USA) and 1% penicillin-streptomycin (Gibco, Carlsbad, CA, USA) under standard culture conditions (humid environment containing 5% CO_2_ and 95% air at 37 °C). To induce differentiation, the culture medium was replaced with DMEM containing 2% horse serum (Gibco, Carlsbad, CA, USA) when the cell confluence reached 80–90%, and the induction period was 7 days. The samples used in the detection of spatio-temporal expression profiles are from Ningxiang boars in four stages (30 days: weaning period; 90 days: nursery period; 150 days: early fattening period; and 210 days: late fattening period), in which Ningxiang pigs were full siblings at the same period and half siblings at different periods. The samples include heart, liver, spleen, lung, kidney, back fat and *longissimus dorsi* muscle (LD). The experimental pigs came from the pig farm of Ningxiang Dalong Animal Husbandry Technology Co., LTD (ChuWeiXiang, Changsha, China).

### 4.2. Plasmid Construction and RNA Interference

The function of circHOMER1 was verified via a gain/loss approach. To construct the overexpression plasmid of circHOMER1, the full-length sequence of circHOMER1 was ligated into pOE3.1-CAHyg vector (JTS, Wuhan, China) through Nhe I/HindIII restriction site. To knock down circHOMER1, SiRNA sequence design uses siDirect (https://sidirect2.rnai.jp/) (accessed on 15 January 2024), and the parameters involved were default. Two siRNAs (si-circHOMER1-1 and -2) and negative control (si-NC) targeting BSJ of circHOMER1 were synthesized from RiboBio Biotechnology Co., Ltd. (Guangzhou, China). The plasmid construction and siRNA of circHOMER1 are described in Table S1.

### 4.3. Cell Transfection

The procedure of cell transfection is as follows: when the confluence of cells reaches 70%, the complete medium is changed to DMEM to culture the cells for 2 h. Transfect and lipofectamine 2000 (Invitrogen, Carlsbad, CA, USA) were incubated with DMEM, respectively; then, they were mixed to form transfection complex, and finally the transfection complex was slowly dropped into cells. After transfection complex was co-cultured with cells for 4–6 h, the culture medium was replaced with complete culture medium to continue cell culture.

### 4.4. RNA Isolation, Reverse Transcription and qPCR

Cells were incubated in a culture medium containing 5 μg/mL actinomycetes D (glpbio, Montclair, CA, USA) or DMSO and collected at a specified time point. Total RNA was extracted from cells and tissues by using TRIzal reagent (Coolaber, Beijing, China). RNA concentration was determined by NanoDrop 2000 (Waltham, MA, USA). Total RNA was incubated with 3 U/μg RNsea R (GENESEED, Guangzhou, China) at 37 °C for 15 min to remove linear RNA, and then we inactivated the enzyme at 70 °C for 10 min for reverse transcription. RNA was reverse transcribed into cDNA by RevertAid First Strand cDNA Synthesis Kit, with DNase I reverse transcription kit (Thermo Scientific, Waltham, MA, USA), and data were collected on the Bio-Rad CFX-96 fluorescence quantitative instrument (Hercules, CA, USA) according to the instructions of TransStart^®^ Top Green qPCR SuperMix kit (Transgen, Beijing, China). The primer details of the target gene are shown in Table S1. GAPDH was a reference gene, and the relative expression of the gene was calculated by 2^−ΔΔct^.

### 4.5. Western Blot Assay

Total protein was extracted by RIPA lysate (Beyotime, Shanghai, China) containing 1% protease inhibitor (Beyotime, Shanghai, China). The concentration of protein was detected by BCA kit (Beyotime, Shanghai, China), and the protein was separated by SDS-PAGE gel (Epizyme Biotechnology, Shanghai, China) and then transferred to PVDF membrane (Millipore, Billerica, MA, USA). After that, the PVDF membrane was incubated with primary antibody at 4 °C overnight and then with secondary antibody at room temperature for 1–2 h. The antibody information was as follows: Anti-CDK4 (1:1000, AiFang, Changshai, China), Anti-PCNA (1:1000, Zenbio, Chengdu, China), Anti-BAX (1:1000, Zenbio, Chengdu, China), Anti-Caspase3 (1:1000, Zenbio, Chengdu, China), Anti-β-actin (1:20,000, TransGen, Beijing, China), Anti-MYOG (1:200, DSHB, Iowa, IA, USA), Anti-MyHC (1:500, DSHB, Iowa, IA, USA), Anti-MyoD (1:1000, Huabio, Hangzhou, China). The protein signal was detected by Meilunbio^®^ fg super-sensitive ECL luminescence reagent (Meilunbio, Dalian, China) according to manufacturer’s instructions, and then the images were obtained using Image Quant LAS 4000 mini chemiluminescence imaging system (GE, Boston, MA, USA). β-actin was used as an internal reference to calculate the relative expression of protein, and the gray value of protein was detected by imageJ software (v1.51j8).

### 4.6. Proliferation Experiment of CCK-8 and EdU

Transfected cells were seeded in 96-well plate, and cell proliferation was detected by CCK-8 reagent (APExBIO, Houston, TX, USA) and EdU cell proliferation kit Alexa Fluor 555 (Meilunbio, Dalian, China). The details of the procedure are in accordance with the manufacturer’s instructions. CCK-8 reagent was added when incubated for 0 h (cell adhesion), 12 h, 24 h and 36 h. The absorbance of 450 nm was collected using microplate reader (Thermo Scientific, Waltham, MA, USA). After the cells were cultured on 96-well plates for 20 h, EdU solution was added, and the proportion of cells labeled by EdU was detected by Axio Vert A1 fluorescence microscope (Zeiss, Oberkochen, Germany).

### 4.7. Immunofluorescence Staining

Cells differentiated for 7 days were washed twice with precooled PBS (Meilunbio, Dalian, China), then fixed with 4% paraformaldehyde (biosharp, Hefei, China) for 15 min, washed with 0.5% triton X-100 (Sigma-Aldrich, St. Louis, MO, USA) for 10 min, and washed with 5% BSA (Biofroxx, Cologne, Germany). The primary antibody (MyHC, 1:200, DHSB, Iowa, IA, USA) was incubated overnight at 4 °C, and the fluorescent secondary antibody (Dylight 649, anti-mouse IgG, 1:1000, Abbkine, Wuhan, China) was incubated for 1 h at room temperature in the dark. The nucleus was stained by DAPI (Solarbio, Beijing, China). Finally, Antifade Mounting Medium (Beyotime, Shanghai, China) was added, and the images were collected using Axio Vert A1 fluorescence microscope (Zeiss, Oberkochen, Germany).

### 4.8. Flow Cytometry Assay

Cell cycle and apoptosis were detected via flow cytometry (Becton Dickinson, Franklin Lakes, NJ, USA). Cells were collected by digesting cells with pancreatin, washed once with precooled PBS, the centrifuged cell precipitate was fixed with 70% ethanol for at least 2 h, and the cell density was 0.2–2 million/mL. And then fixed cells were stained with Cell Cycle and Apoptosis Analysis Kit (Meilunbio, Dalian, China). Cells were collected by digesting cells with trypsin, washed once with precooled PBS, and resuspended with precooled PBS after centrifugation, with a cell density of 1 million/mL. The cells were filtered with a 200-mesh sieve, and 100 microliters of cell suspension was stained with Annexin V-FITC/PI cell apoptosis detection kit (Meilunbio, Dalian, China). The test must be completed within 2 h. The detailed staining procedure was carried out according to the instructions. The data were further analyzed by FLowjo software v9 (TreeStar, Ashland, OR, USA).

### 4.9. Subcellular Fractionation and RNA Extraction

RNA was extracted from the nucleus and cytoplasm of cells and muscle tissues using PARIS^TM^ Kit following the manufacturer’s guidelines (Invitrogen, Carlsbad, CA, USA). The formula for calculating the nuclear–cytoplasmic relative ratio of circRNA was as follows: RNA_cyto_ Ratio of circHOMER1 = 2^−CTcyto^/(2^−CTnuc^ + 2^−CTcyto^), RNA_nuc_ Ratio of circHOMER1 = 1 − RNA_cyto_ Ratio.

### 4.10. Fluorescence In Situ Hybridization (FISH)

Then, 20 nt was taken from the upstream and downstream of the back splicing junction (BSJ) site of circRNA, and pair ends of the probe were labeled with Cy3, which was stained on cell slides and muscle tissue sections. The probe signal was detected with fluorescence in situ hybridization kit following the manufacturer’s guidelines (BOSTER, Pleasanton, CA, USA). Photographs were collected using fluorescence microscope and scanner. circHOMER1 fluorescent probe was synthesized from Boster Biotechnology Co., Ltd. (Pleasanton, CA, USA). FISH probe sequences are shown in Table S1.

### 4.11. Prediction of Protein Coding Ability of circRNA

The identified circRNA sequence was blasted in UCSC (https://genome.ucsc.edu/cgi-bin/hgBlat?Command=start) (accessed on 15 November 2023) to obtain the host gene, exon number and length of circRNA. The coding ability of circRNA was predicted by ORF [37], IRES [38] and circRNADb. After the sequence of circRNA was repeated once, it was copied into ORF (https://www.ncbi.nlm.nih.gov/orffinder/) (accessed on 15 November 2023) for prediction. As a result, the ORF across back splicing sites was selected. Search circRNAb (http://reprod.njmu.edu.cn/CGI-bin/circrnab/brogene_front.php) (accessed on 15 November 2023) by “advance retrieval/browse by gene symbol”, then determine circRNA according to the size of the sequence, and check whether it has ORF and IERS sites; the threshold of ORF was set as R score ≥ 1.5. The protein coding potential of circRNA was predicted by CPAT online website (http://lilab.research.bcm.edu/) (accessed on 15 November 2023) [39].

### 4.12. Homology Transformation of circRNA and Conservation Analysis of miRNA

Homologous transformation between species was carried out by detecting the conservativeness of circRNA. Because the information of human circRNA is more comprehensive, in this study, the circRNA obtained by sequencing in pigs was transformed into human circRNA ID by sequence and length, and then homologous transformation was carried out. Homologous conversion is realized through online databases circBank (http://www.circbank.cn/searchCirc.html) (accessed on 15 November 2023) and circAtlas 3.0 (https://ngdc.cncb.ac.cn/circatlas/index.pHp) (accessed on 15 November 2023). According to the name and length information of host gene, circRNA is determined in circBank. The circbank ID obtained in circRNADb is converted into circAtlas ID in circAtlas 3.0, and then whether circHOMER1 has the same circRNA in mice and pigs was checked.

The mature sequence (CGCAUCCCCUAGGGCAUUGGUGU) of ssc-miR-324-5p was obtained in mirBase (https://mirbase.org/search/) (accessed on 15 November 2023), and the corresponding miRNAs in humans and mice were searched according to the sequence. The conservative standard was that the seed region (2–8 nt at the 5′ end) was completely consistent.

### 4.13. Double Luciferase Report Assay

To construct luciferase reporter plasmid, we synthesized the fragment and mutation sequence of circRNA combined with miR-324-5p and then inserted them into pmirGLO vectors (pmirGLO-circHOMER1 and pmirGLO-MUT). For the reported experiment, we transfected pmirGLO-circHOMER1 and miR-324-5p mimics or mimics NC into HEK-293T cells. At 48 h after transfection, cells were lysed and luciferase activity level was detected by double luciferase reporting assay system (Promega, Madison, WI, USA). Firefly luciferase activity level was normalized to Renilla luciferase activity levels.

### 4.14. Statistical Analysis

All results are presented as “mean ± standard deviation (SD)”. Two-tailed *t*-test with SPSS 22 software (IBM, Armonk, NY, USA) was used to detect the difference between groups, and Origin software was used to visualize the data, with *p* < 0.05 and *p* < 0.01 set as the difference significance level.

## 5. Conclusions

CircHOMER1 is an exon circRNA with a length of 352 nt, which consists of three exons. It promotes myoblast proliferation and apoptosis and inhibits myoblast differentiation. CircHOMER1 may participate in myoblast development through the ceRNA regulatory network and coding small peptides. These results provide a reference for the in-depth analysis of skeletal muscle development and molecular breeding of Ningxiang pigs.

## Figures and Tables

**Table 1:** Homology transformation of circHOMER1.

Source	ID
circRNAdb	hsa_circ_22696
circbank	hsa_circHOMER1_003
circAtlas3.0	hsa-HOMER1_0008
Basic information	
circBank ID: hsa_circHOMER1_003	Host gene Symbol: HOMER1
circBase ID: hsa_circ_0073132	bestTranscript: NM_004272
Position: chr5: 78742875-78752841 strand: -	Annotation: ANNOTATED, CDS, coding, INTERNAL, OVCODE, OVEXON
Length: 382	
RNA sequence:	
GGAACAACCTATCTTCAGCACTCGAGCTCATGTCTTCCAAATTGACCCAAACACAAAGAAGAACTGGGTACCCACCAGCAAGCATGCAGTTACTGTGTCTTATTTCTATGACAGCACAAGAAATGTGTATAGGATAATCAGTTTAGATGGCTCAAAGGCAATAATAAATAGTACCATCACCCCAAACATGACATTTACTAAAACATCTCAGAAGTTTGGCCAGTGGGCTGATAGCCGGGCAAACACCGTTTATGGATTGGGATTCTCCTCTGAGCATCATCTTTCGAAATTTGCAGAAAAGTTTCAGGAATTTAAAGAAGCTGCTCGACTAGCAAAGGAAAAATCACAAGAGAAGATGGAACTTACCAGTACACCTTCACAG	
conserved mm9 circRNA: >mmu_circ_0004882	
GGAGCAACCTATCTTCAGCACTCGAGCTCATGTCTTCCAGATTGACCCGAACACAAAGAAGAACTGGGTACCCACCAGCAAGCATGCAGTTACTGTATCTTATTTTTATGACAGCACAAGAAATGTGTATAGGATAATCAGTTTAGATGGCTCAAAGGCAATAATAAATAGCACCATCACACCAAACATGACATTTACTAAAACATCTCAAAAGTTTGGCCAATGGGCTGATAGCCGGGCAAACACTGTTTATGGACTGGGATTCTCCTCTGAGCATCATCTTTCAAAATTCGCAGAAAAGTTTCAGGAATTTAAGGAAGCTGCTCGGCTTGCAAAGGAGAAGTCGCAGGAGAAGATGGAGCTGACCAGTACCCCTTCACAG	
coding_potential_assessment	
circBank ID: hsa_circHOMER1_003	Fickett_score: 0.8529
circRNA_size: 1146	Hexamer_score: 0.0431
ORF_size: 240	coding_prob: 0.1187

## Data Availability

Data are contained within the article and Supplementary Materials.

## Supplementary Materials

The following supporting information can be downloaded at: https://www.mdpi.com/article/10.3390/ijms26136264/s1.
