# Supplementary figures and images for: Function and Molecular Mechanism of Circhomer1 in Myogenesis
Source: Int J Mol Sci. 2025 Jun 28;26(13):6264. doi: 10.3390/ijms26136264 (PMC12249824; doi:10.3390/ijms26136264)

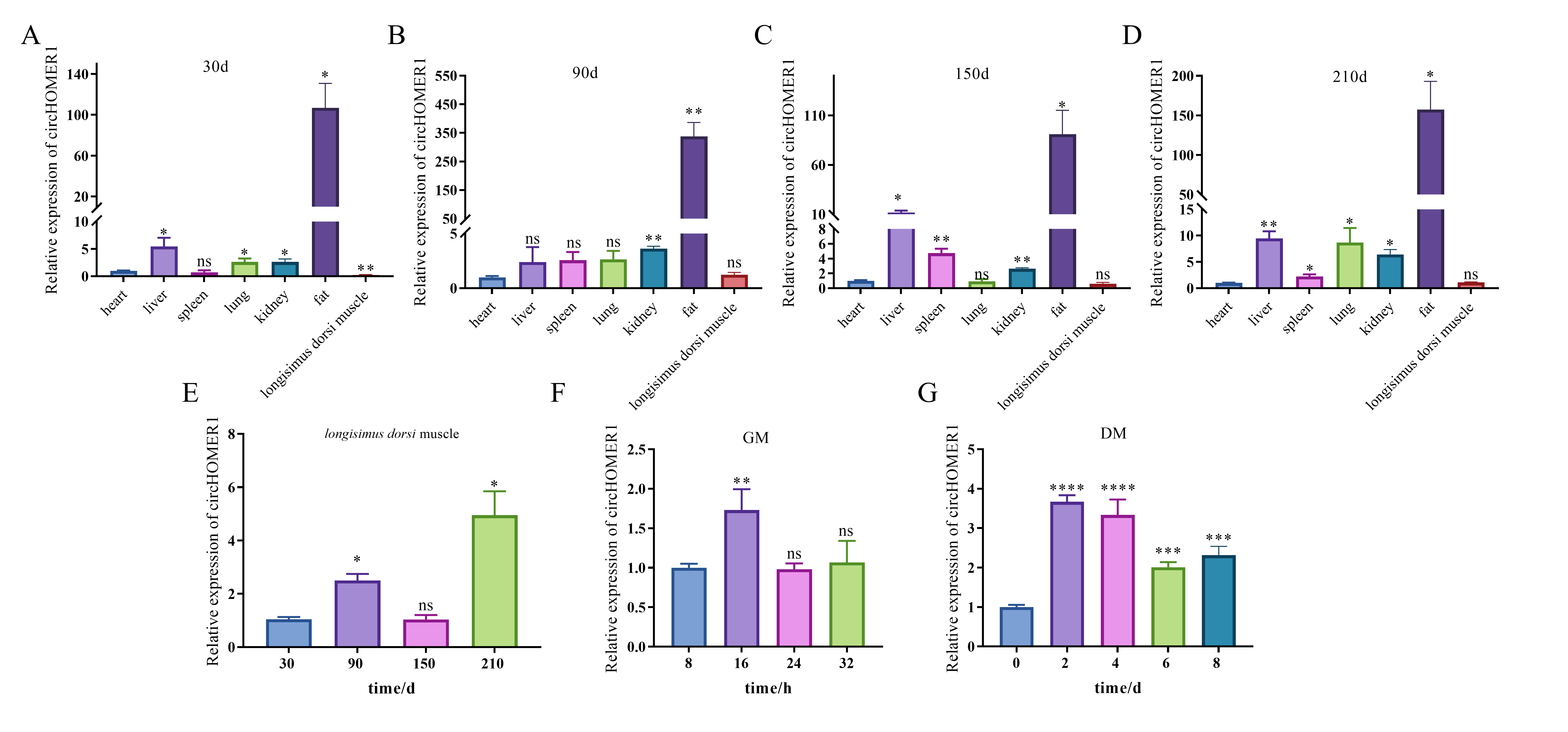

Supplement: Supplementary file 1 [file ijms-26-06264-s001.zip › Fig. S1 expression pattern.tif]

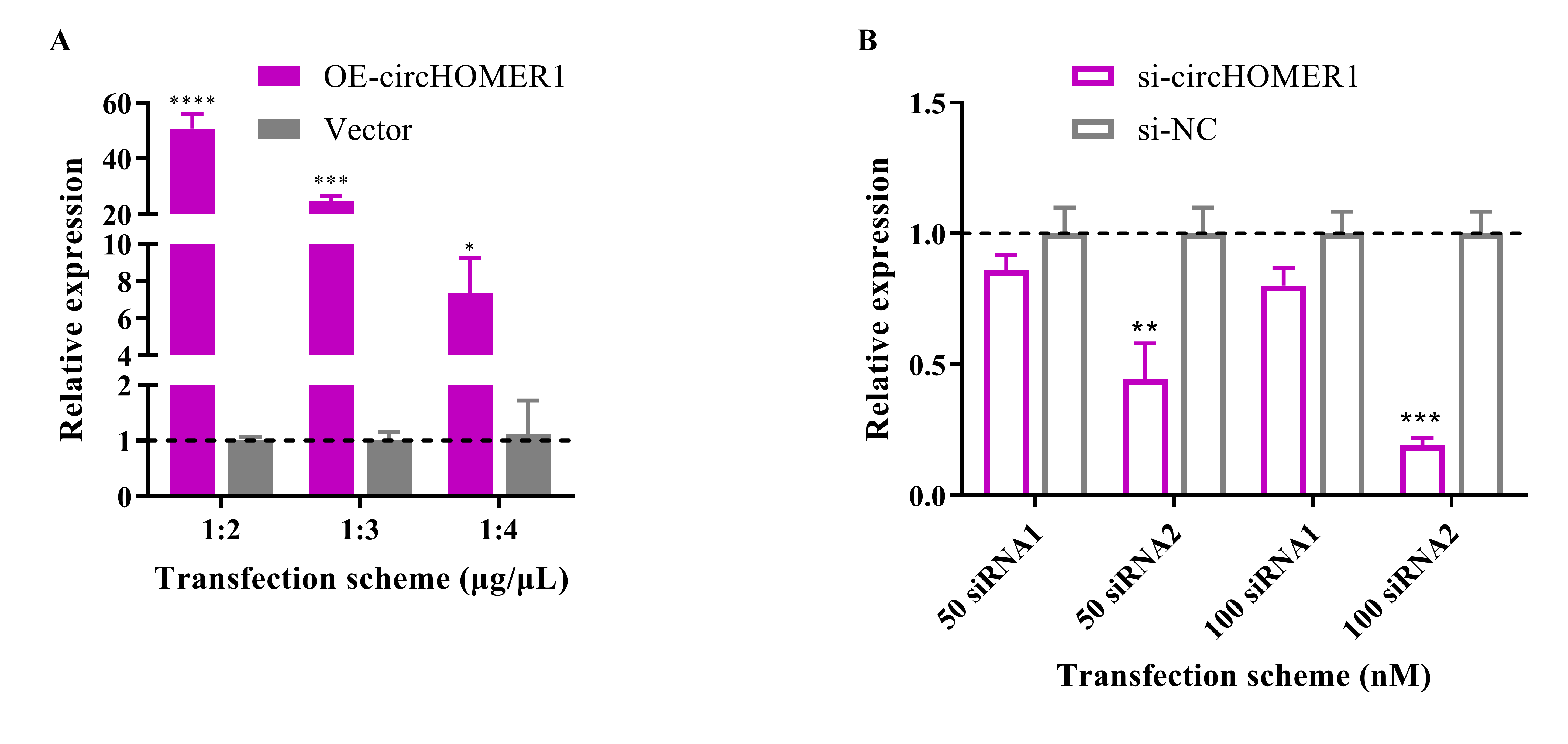

Supplement: Supplementary file 1 [file ijms-26-06264-s001.zip › Fig. S2 Transfection efficiency.tif]
